# Differences in trauma history and psychopathology between PTSD patients with and without co-occurring dissociative disorders

**DOI:** 10.3402/ejpt.v4i0.21452

**Published:** 2013-11-26

**Authors:** Pascal Wabnitz, Ursula Gast, Claudia Catani

**Affiliations:** 1 Department of Psychology, University of Bielefeld, Bielefeld, Germany; 2 Private Praxis, Dammholm, Germany

**Keywords:** Dissociation, dissociative disorders, DDNOS, PTSD, structural dissociation

## Abstract

**Background:**

The interplay between different types of potentially traumatizing events, posttraumatic symptoms, and the pathogenesis of PTSD or major dissociative disorders (DD) has been extensively studied during the last decade. However, the phenomenology and nosological classification of posttraumatic disorders is currently under debate. The current study was conducted to investigate differences between PTSD patients with and without co-occurring major DD with regard to general psychopathology, trauma history, and trauma-specific symptoms.

**Methods:**

Twenty-four inpatients were administered the Clinician-Administered PTSD Scale for DSM-IV (CAPS) and the Mini-Structured Clinical Interview for DSM-IV Dissociative Disorders (MINI-SKID-D) to assess DD and PTSD. Additionally, participants completed questionnaires to assess general psychopathology and health status.

**Results:**

Symptom profiles and axis I comorbidity were similar in all patients. Traumatic experiences did not differ between the two groups, with both reporting high levels of childhood trauma. Only trauma-specific avoidance behavior and dissociative symptoms differed between groups.

**Conclusion:**

Results support the view that PTSD and DD are affiliated disorders that could be classified within the same diagnostic category. Our results accord with a typological model of dissociation in which profound forms of dissociation are specific to DD and are accompanied with higher levels of trauma-specific avoidance in DD patients.

Since Pierre Janet described the phenomenon of dissociation at the beginning of the 20th century (Janet, 1889), research on dissociation has led to the formulation of comprehensive theories addressing the development of pathological and normal forms of dissociation (for an overview see Dell & O'Neil, 2009). While it has been proposed that the actual link between traumatic stress and dissociation is weak (e.g., Giesbrecht, Lynn, Lilienfeld, & Merckelbach, 2008, 2010), others refute this claim pointing to a clear and persistent association between traumatic stress and dissociation (Bremner, 2010). Until now, the relationship between traumatic stress and dissociation that has been supported by a vast body of literature (e.g., Carlson, Dalenberg, & McDade-Montez, 2012; Nijenhuis & Van der Hart, 2011) remains controversial.

In general, the link between dissociative symptoms and PTSD has been studied in various populations (e.g., Briere, Scott, & Weathers, 2005; Koopman, Classen, & Spiegel, 1994) whereby dissociation has been shown to be a strong predictive factor for PTSD. People who report dissociative symptoms in the aftermath of and during a traumatic event are at higher risk for developing PTSD compared to those without dissociative symptoms (Fullerton et al., 2000). Furthermore, several authors have claimed that there is an association between severity of dissociative and posttraumatic symptoms (Branscomb, 1991; Putnam, 2003) and that chronic dissociation is a stronger predictor for PTSD than peritraumatic dissociation (Briere et al., 2005). However, even if considering recent research the specific relationship and interplay between traumatic experiences, dissociation and PTSD remains controversial. For this reason, we will briefly introduce different etiological theories, each postulating specific links among trauma, dissociation, and its disorders.

Considering a psychobiological perspective on trauma, Schauer and Elbert (2010) recently suggested that there might be two evolutionary primed reaction tendencies to reminders of trauma in PTSD. One is thought to be characterized by an activation of the sympathetic nerve system that leads to typical PTSD symptoms, such as flashbacks or hyperarousal. The other reaction pattern is supposed to be associated with a parasympathetical inhibition of sensory and functional systems, that is, a dissociative response (Schauer & Elbert, 2010). This view of different reaction tendencies to trauma reminders in PTSD is consistent with the notion of separated neural manifestations of dissociative and hyperarousal subtypes of PTSD put forward by Lanius and colleagues (Lanius et al., 2010). According to Schauer and Elbert, when dissociation was the prominent peritraumatic response, a comparable dissociative reaction might reappear when the traumatic memory is reactivated. Against the background of a dimensional understanding of dissociation, the suggestions made by Schauer and Elbert (2010) might be applicable not only to dissociative symptoms but also to complex dissociative disorders (DD). The assumption would be that the recurring appearance of the conditioned peritraumatic dissociation will gradually become a chronic disorder and can finally be perceived as a permanent personality change with a dissociative identity disorder (DID) being the most extreme form. This idea is in part consistent with the theory by Van der Hart and colleagues (e.g., Nijenhuis, Van der Hart, & Steele, 2002; Steele, Van der Hart, & Nijenhuis, 2009; Van der Hart, Nijenhuis, & Steele, 2006) who postulate that dissociative parts of traumatic experiences become increasingly fragmented, therefore more chronic and habituated the longer the traumatic experience persists. The authors note that this mechanism is one factor in the development of structural dissociation of the personality, a condition that can lead to the development of a DID. Most notably, the latter conceptualization argues for a different perspective on trauma-related disorders, in the sense that PTSD should be seen as DD (Steele et al., 2009).

Looking further at posttraumatic theories of DD, several risk factors for chronic dissociation (e.g., intense and repetitive maltreatment in early childhood; Gleaves, 1996; Putnam, 2003) have been proposed. A cross-sectional study showed that patients who experienced multiple traumata reported more dissociation than single-trauma patients (Hagenaars, Fisch, & van Minnen, 2011). Moreover, almost 95% of patients with DID fulfill DSM-IV criteria for PTSD (Rodewald, Wilhelm-Gössling, Emrich, Reddemann, & Gast, 2011b).

As mentioned above, the latest developments in posttraumatic theories, namely the theory of structural dissociation, (Steele et al., 2009) postulate three levels of complexity in dissociation of the personality (Van der Hart, Nijenhuis, Steele, & Brown, 2004). This structural dissociation is thought to be maintained by learning principles, such as classical conditioning, evaluative conditioning, and social learning processes (Van der Hart, Nijenhuis, & Steele, 2005).

Regarding the relationship between complex DD and PTSD, one core assumption of the theory of Van der Hart and colleagues (Van der Hart et al., 2005) is that trauma-related disorders (e.g., PTSD, complex PTSD, acute stress disorder [ASD] and DD) can be arranged on a continuum ranging from simple forms of PTSD and ASD to complex PTSD and DID. Although they emphasized the commonalities between various trauma-related disorders, the authors presume that there are differences to be found in both the symptoms and trauma history in these disorders (Steele et al., 2009) and suggest “…that severity of structural dissociation provides an important organizing principle for classifying posttraumatic disorders” (p. 247). In conclusion, the relationship among traumatic stress, dissociation, and related disorders can be illuminated from different perspectives, leading to various assumptions. The current version of the DSM (DSM-V; APA, 2013) considers some of the ideas reviewed so far in its reformulation of PTSD by including a dissociative subtype which has repeatedly been identified in the recent research as reviewed by Dalenberg and colleagues (Carlson et al., 2012; Dalenberg & Carlson, 2012). In support of this, current neuroimaging findings have shown that such a subtype, comprising symptoms of depersonalization and derealization, is evident in healthy as well as in clinical samples (Lanius, Brand, Vermetten, Frewen, & Spiegel, 2012; Steuwe, Lanius, & Frewen, 2012). Moreover, those patients with the dissociative subtype of PTSD show a distinct pattern of neurobiological responses (Lanius et al., 2010) and seem to benefit differently from current treatments (Lanius et al., 2012). In particular, such important clinical implications highlight the need to further disentangle the complex interplay of traumatic stress, dissociation, and related disorders.

To our knowledge, no study to date has investigated how PTSD patients differ from patients with a DD regarding symptom complexity, trauma history, and additional comorbid psychiatric disorders. Therefore, the aim of the present study was to assess variables related to trauma history and a variety of clinical characteristics in a sample of PTSD patients with and without co-occurrent DSM-IV DD. As the vast majority of patients with DD have a co-occurring PTSD (Rodewald et al., 2011b) we were not able to recruit a sample of patients with only DD but without a PTSD. Considering this and the limited sample size of our groups, the present study should be taken as a pilot attempt to compare patients with PTSD only to patients with comorbid DD.

In accordance with Steele and colleagues (2009) and based on the reviewed literature, we hypothesized that a difference will be found between patients with PTSD and patients with additional DID in terms of symptom complexity, trauma history, and comorbid disorders. These predictions in general are in accordance with the theories and recent research contributions reviewed above. Both Schauer and Elbert (2010) as well as Van der Hart and colleagues (e.g., Nijenhuis et al., 2002; Steele et al., 2009; Van der Hart et al. 2006) dissociation should be linked to a distinct pattern of symptoms and associated predictors such as trauma history and comorbid disorders.

## Method

### Participants

Participants were 24 patients of an inpatient clinic in Bielefeld, Germany. Consecutively admitted patients were evaluated during a period of 3 months. All patients were comparable regarding the amount of treatment and time since admission to the clinic and between 18 and 60 years. Exclusion criteria were established by the clinic (current psychotic disorder, acute substance abuse or dependence, acute suicide intention or ideation, age <18 years). Our aim was to assess subjects with a history of traumatic life experiences and either (1) PTSD without any comorbid DD, (2) PTSD with comorbid DD, and (3) DD without PTSD. Due to the specialization for trauma-related disorders, every patient who was administered to the study within the period of assessment presented PTSD. Hence, it was not possible to recruit a “dissociation-only” group.

Participants were divided into two subgroups on the basis of structured clinical interviews. The “DD” group consisted of patients with a DSM-IV diagnosis of at least one major DD (DID or DD not otherwise specified [DDNOS] and PTSD. The other group included those patients who received the diagnoses of a current PTSD as described in the DSM-IV but without a comorbid diagnosis of any major DD (except of *dissociative amnesia* as it is a very common condition in PTSD patients) and were labeled “PTSD group.” The diagnoses in both subgroups are shown in Table 1.

**Table 1:** Socio-demographic characteristics and diagnosis of 24 patients with and without a dissociative disorder

	Trauma subgroup				
		
	DD (*n*=13)		PTSD (*n*=11)		Total (*N*=24)
		
Characteristics	*n*	%	*n*	%	*N*
Sex male^a^	2	15.4	1	9.1	3
Social support^a^					
Good	2	15.4	1	9.1	3
Sufficient	2	15.4	2	18.2	4
Insufficient	8	61.5	5	45.5	13
Bad	1	7.7	3	27.3	4
Single, divorced, widowed, or separated^a^	8	61.5	7	63.6	15
Education^a^					
< High school	2	15.4	4	36.4	6
High school or equivalent	6	46.1	3	27.3	9
Some college or technical	0	0	2	18.2	2
College graduation	5	38.5	2	18.2	7
Diagnosis					
Dissociative identity disorder	8	61.5	0	0	8
DDNOS	5	38.5	0	0	5
PTSD	13	100	11	100	24
	Mean	SD	Mean	SD	Mean
Age	37.15	12.32	33.00	10.38	35.25

a No between-group differences were statistically significant. Analyses were conducted using Mann–Whitney *U* tests, and Chi-square test.

### Procedure

All participants provided written informed consent after the aims and procedure of the study was explained to them. Participants filled out the questionnaires within 2 days of being admitted to the clinic and were interviewed by an experienced clinician. Interviews were carried out by one of the authors (P. W.) during two sessions on consecutive days within the first 2 weeks of admission. In the first session, the Clinician-Administered PTSD Scale (CAPS; Blake et al., 1995) and the MINI International Neuropsychiatric Interview (MINI, Sheehan et al., 1998) were carried out, whereas the mini-SCID-D (Structured Clinical Interview for DSM-IV Dissociative Disorders; SCID-D, Steinberg, 1994) was administered during the second session. To ensure validity of the assessment, all interviews were videotaped for supervision purposes. The University of Bielefeld Ethic Committee approved the study protocol.

### Measures

#### Socio-demographic and clinical variables

Participants were asked a standardized set of questions related to socio-demographic information, such as socio-economic status, education. In addition, the interview contained several questions about prior therapeutic experiences, the number and duration of prior therapies, the duration of incapacity for work due to mental health problems, and quality of life.

#### Axis I disorders

The MINI (Sheehan et al., 1998) was used to assess current psychiatric DSM-IV axis I diagnoses.

#### Childhood trauma

The German version (Wingenfeld et al., 2010) of the 28-item Childhood Trauma Questionnaire (CTQ; Bernstein & Fink, 1998) was carried out as part of the structured interview to assess the severity and frequency of childhood abuse. The CTQ measures five areas of child maltreatment: emotional abuse, physical abuse, sexual abuse, physical neglect, and emotional neglect. In addition, the clinical interview contained some questions related to participants’ trauma history (e.g., duration of abuse) such as: “If you have ever experienced a traumatic event, was it a single or an ongoing/repetitive event?; “how long lasted the worst ongoing event?”

#### Posttraumatic stress disorder

The Clinician-Administered PTSD Scale for DSM-IV (CAPS; Blake et al., 1995) was used for the diagnosis and quantification of PTSD.

#### Dissociative symptoms

Dissociative symptoms were assessed using the German version of the MINI-SCID-D (Gast & Rodewald, 2004). The Mini-SCID-D is a clinician-administered interview that evaluates the severity and frequency of five core symptoms of DD (depersonalization, derealization, amnesia, identity confusion, and identity alteration). The original version has shown excellent psychometric properties in a wide variety of international surveys (Ross, 1997; Steinberg, 2000). Participants also filled out the Fragebogen für Dissoziative Symptome (FDS: Spitzer, Michels, Siebel, Gänsicke, & Freyberger, 2004), the German version of the Dissociative Experience Scale (DES; Bernstein & Putnam, 1986) at the time of admission to the clinic. The FDS is a self-report instrument assessing the severity of a range of dissociative symptoms. We also included the Multidimensional Inventory of Dissociation (MID; Dell, 2006, German version Gast, 2003), a self-report instrument that explicitly assesses the phenomenological domain of pathological dissociation, as opposed to symptoms of “normal” dissociation, such as absorption (for further details on this debate see Rodewald, Dell, Wilhelm-Gössling, & Gast, 2011a).

#### Depressive symptoms

The Beck Depression Inventory (BDI, Beck & Steer, 1987) was used to assess the severity of depressive symptoms.

#### Somatoform symptoms

Participants filled out the Screening for Somatoform Symptoms-7 (SOMS-7; Rief & Hiller, 1997) in order to determine severity and number of somatoform symptoms (e.g., pain, diarrhea, headache) during the last 7 days.

All measures used in this study were in German.

### Data analysis

All statistical analyses were carried out using the “Statistical Package for the Social Sciences” SPSS 13. Due to the small sample size, data analyses were carried out using non-parametric statistics. All data met the required assumptions. To compare means and frequencies of the variables between groups, Mann–Whitney *U* tests were used for continuous variables, whereas Chi-square tests were used for categorical variables. Due to the small sample size between-group effect sizes are presented as Hedge's *g*.

## Results

### Sample characteristics

Patients with and without DD did not significantly differ on any demographic characteristics (Table 1). A group comparison related to clinical characteristics is shown in Table 3.

In general, the lack of differences between groups must be taken with caution as the very sample size in each group may have increased the probability of type II error.

### Child abuse and other traumatic experiences

Table 2 provides an overview of group comparisons regarding participants’ trauma history. There were no significant differences between patients with PTSD only, and those with a co-occurring DD regarding any type of childhood abuse or the number of traumatic experiences throughout their life. The age of onset of childhood trauma was also similar in both groups. The only significant group differences were related to the frequency and duration of potentially traumatizing events. DD patients more often reported ongoing traumatic experiences in the past whereas, PTSD patients did not χ^2^(1, *n*=24) = 6.29, *p*<0.001, phi = − 0.52. Moreover, a distinct difference was found regarding “duration of the worst event” showing that subjects in the DD group reported a longer duration compared to the PTSD-only group (Hedge's *g*=1.21).

**Table 2:** Trauma characteristics for PTSD patients with (DD) and without (PTSD) dissociative disorder

	Trauma subgroup				
				
	DD (*n*=13)	PTSD (*n*=11)	Analysis^a^		Effect size (*r*)
	
	Median	Median	*U*	*p*	0.19
Number of traumatic life events	6.0	6.0	7.0	0.31	0.08
Age at first trauma (years)	4.5	4.0	49.5	0.97	0.54
Duration of worst event (month)	64.0	12.0	11.0	0.15	0.12
CTQ_emotional_ _neglect_	22.0	22.0	62.0	0.58	0.00
CTQ_physical_ _neglect_	12.0	14.0	63.0	0.62	0.12
CTQ_sexual_ _abuse_	15.0	10.0	59.0	0.92	0.33
CTQ_emotional_ _abuse_	21.0	17.5	32.0	0.11	0.11
CTQ_physical_ _abuse_	11.5	11.5	50.0	0.51	0.19

a Results are from between-subject Mann–Whitney *U* tests; effect size displayed as *r*.

### PTSD diagnoses and severity

**Fig. 1:**
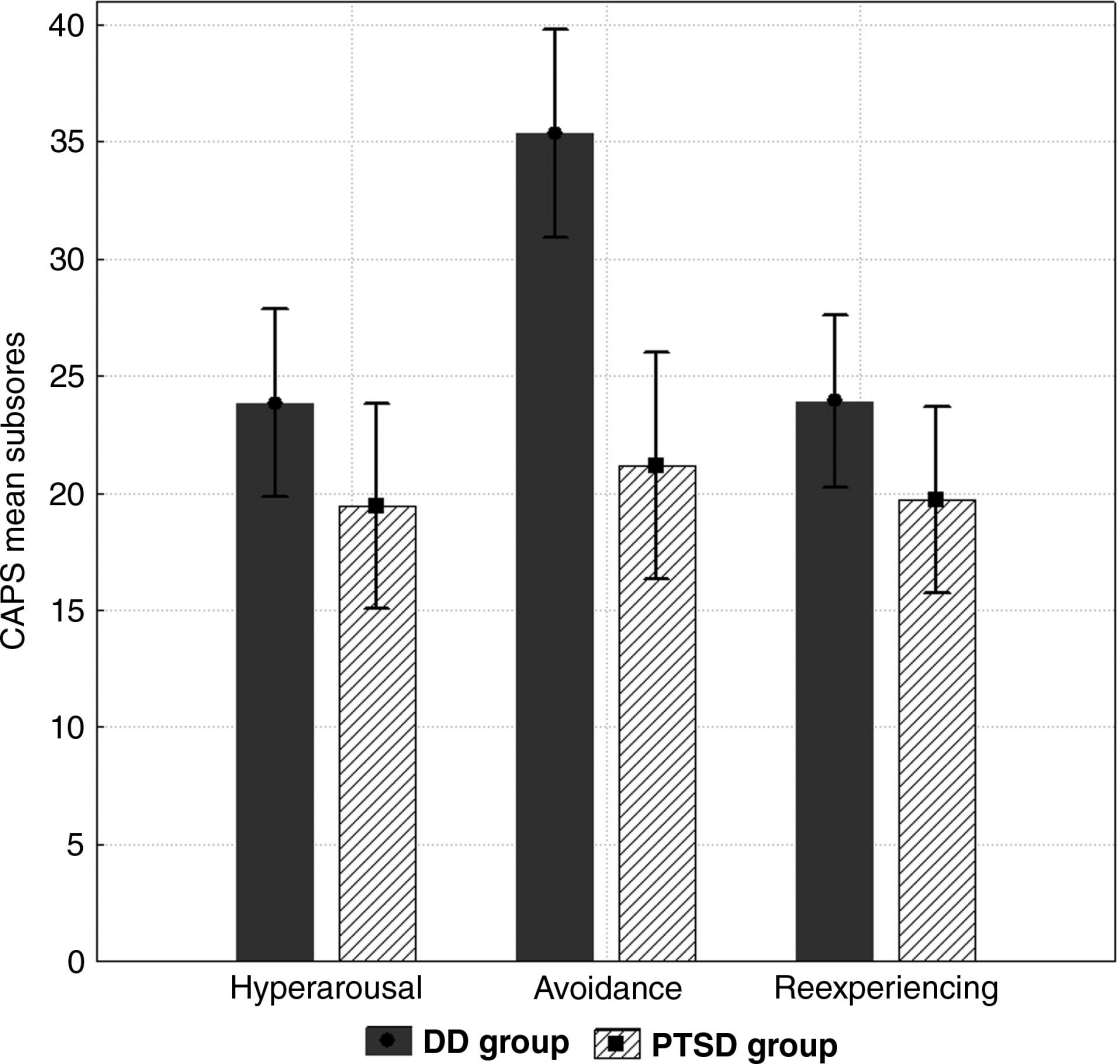
CAPS subscores (mean) for PTSD patients with and without dissociative disorder.

All participants met DSM-IV criteria for current PTSD. Patients within the PTSD group had a mean CAPS score of 60.36, whereas the DD group had a mean CAPS score of 80.33, *t*(18) = 3.17, *p*<0.001. Figure 1 shows group means for the three CAPS subscales (avoidance, re-experiencing, hyperarousal). The DD group had a significantly higher score than the PTSD group on the avoidance subscale (DD_=_35.00, SD_DD_=8.54 vs. *M*_PTSD_=21.18, SD_PTSD_=7.96, *F*(1,18) = 13.97, *p*< 0.005), whereas there were no significant differences with respect to re-experiencing and hyperarousal (Fig. 1).

### Dissociative symptoms and other psychopathology

On the FDS, the DD groups did not report more dissociative symptoms than the PTSD group whereas on the MID, the DD group reported significantly more dissociative symptoms than the PTSD group. Group differences on the BDI and SOMS were examined by means of an ANOVA. As shown in Table 3, there were no significant differences between the DD and the PTSD group. In addition, Chi-square tests revealed no differences with respect to the frequencies of DSM-IV axis one disorders as diagnosed with the MINI, neither in the section of mood disorders χ^2^(1, *n*=24) = 0.07, *p*=0.80, phi = 0.06, substance use disorders χ^2^(1, *n*=24) = 1.16, *p*=0.28, phi = 0.24, psychotic disorders (none), eating disorders χ^2^(1, *n*=24) = 1.16, *p*=0.28, phi = 0.24, or anxiety disorders χ^2^(1, *n*=24) = 0.40, *p*=0.53, phi = 0.14. The mean number of MINI diagnoses did not differ significantly between the two groups.

**Table 3:** General psychopathology and prior psychotherapy for PTSD patients with (DD) and without (PTSD) dissociative disorder

	Trauma subgroup				
				
	DD (*n*=13)	PTSD (*n*=11)	Analysis^a^		Effect size
	
	Median	Median	*U*	*p*	*r*
General psychopathology					
Number of axis I disorders	2.0	2.0	36.5	0.31	0.21
BDI score	28.0	26.0	64.0	0.66	0.17
FDS score	36.0	27.0	49.0	0.19	0.30
MID score	149.0	54.0	20.0	<0.01	0.76
SOMS score	19.4	16.7	48.0	0.27	0.17
Prior psychotherapy					
Total number of treatments	1.0	1.0	12.5	<0.05	0.72
Duration (month)	2.5	1.0	11.0	<0.05	0.47
Duration of incapacity to work	3.0	0.5	3.0	<0.05	0.45

a Results are from between-subject Mann–Whitney *U* tests; effect size displayed as *r*.

### Duration of prior therapy

Mann–Whitney *U* tests revealed that the DD group had received more psychotherapeutic treatments during the past 10 years and that the duration of the intervention was longer than in the PTSD group (see Table 3).

## Discussion

The goal of the present study was to examine differences between PTSD patients with and without comorbid DD. As noted before, because of the small sample sizes, the present results have to be taken with caution and cannot be easily generalized. Having this limitation in mind, we still think that our findings are important and might stimulate further research with larger patient groups. A central result of our study was that the two groups of patients were very similar with respect to their general trauma history and comorbid disorders. However, patients with comorbid DD reported more symptoms of pathological dissociation (MID domains) had more frequent therapeutic experiences and reported longer periods of incapacity for work due to mental health problems. In addition, even though both groups did not differ with respect to overall PTSD severity, patients with comorbid DD had a higher score on trauma-related avoidance.

In general, our findings are consistent with current etiological theories regarding the differentiation between PTSD and DD. Given the impact of various childhood traumata on the pathogenesis of psychopathology (i.e., Egeland, 2009; Risch et al., 2009) and the relationship among childhood trauma, PTSD, (i.e., Breslau, 2002) and DD (for a review see Van Ijzendoorn & Schuengel, 1996) one might consider similar etiological pathways, even though it is suggested that DD rarely occurs as a result of single traumatic events such as motor vehicle accidents while PTSD probably does. In line with recent developments reformulations of the PTSD section in the DSM-V (APA, 2013), it seems evident that both disorders share a common etiology. However, prior work has failed to disentangle fundamental differences and similarities between patients with DD and PTSD patients regarding childhood trauma and general psychopathology. Unfortunately, the present study was limited by the fact that we could not include patients with DD but without co-occurring PTSD. Since PTSD is highly prevalent in most DID patients (see also Rodewald et al., 2011b) this group might be hard to find. However, consistent with prior research (for an overview see Waelde, Silvern, Carlson, Fairbanks, & Kletter, 2009) we found a high prevalence of dissociative symptoms both in the PTSD group as well as in patients with comorbid DD. Recently, Rodewald and colleagues (2011b) showed high overlaps in comorbidity profiles and total number of DSM-IV axis I diagnoses among DID, DDNOS, and PTSD patients. Our results support these findings and provide further evidence for the existence of a comparable symptom profile of patients in the aftermath of traumatic experiences. This has implications for the debate surrounding the link among PTSD, DDNOS and DID and the basic hypothesis proposed by Nijenhuis and colleagues (2002). Whereas levels of dissociation, assessed with the FDS, were not significantly higher in PTSD patients with a DD compared to the group with PTSD only, the domains of dissociation measured with the MID led to higher scores in patients with DD. The MID was developed to explicitly measure pathological symptoms of dissociation (Dell, 2006). Our results indicate that differences between PTSD patients with and without DD emerge particularly with respect to rather severe levels of dissociation, as opposed to “normal” dissociative features such as absorption (generally referred to as the most common dissociative symptom). In fact, Rodewald and colleagues (2011b) also found significantly higher MID scores in DD patients compared to non-dissociative (including PTSD) patients. Our findings extend those of Rodewald and colleagues, suggesting a typological model of pathological dissociation (Rodewald et al., 2011b), involving profound differences in experienced and reported dissociation between DD and other psychiatric patients. While “normal” dissociation can be mostly explained by absorption and “alterations of consciousness” (Nijenhuis & Van der Hart, 2011) dissociative symptoms reported by DD patients might be qualitatively different.

Our finding that the groups did not differ with respect to type and frequency of traumatic experiences questions the predictive power of these factors in the distinction of both disorders. Whereas several authors suggested that more severe early childhood trauma would be reported in DID or DDNOS compared to PTSD patients (i.e., Steele et al., 2009; Waelde et al., 2009), our study failed to find substantial differences with respect to severity of childhood abuse when measured with a standardized instrument. However, chronicity of abusive experiences in childhood may be key as PTSD patients with DID and/or DDNOS in our study more often reported chronic trauma in their early childhood than patients without a DD. This is in line with Hagenaars and colleagues (Hagenaars et al., 2011) stating that the experience of multiple/chronic trauma was associated with higher rates of dissociation in a sample of PTSD patients. However, this result has to be interpreted with caution since we assessed chronicity and frequency with just one single item. Again, the small sample size in the current study limits the significance and generalizability of this finding. Thus, replications with future studies investigating larger patient populations with more elaborate trauma measures (see also Nijenhuis, Spinhoven, Van Dyck, Van der Hart, & Vanderlinden, 1998) are warranted.

A careful interpretation of the results of the present study could be that the chronicity of traumatic experiences might be more important than the trauma type in the development of DD and PTSD. Compared to patients with PTSD only, those with co-occurring DD reported significantly more trauma-specific avoidance behavior as assessed by the CAPS (Blake et al., 1995), whereas there were no differences with respect to the other PTSD symptom clusters (intrusions and hyperarousal). In accordance with this result, Steele and colleagues (2009) have already noted that avoidance of trauma-related cues is one core feature in the etiology of DID and/or DDNOS. Furthermore, Waelde and colleagues (2009) suggest that “chronic dissociation may become a habitual response to relatively lower magnitude stressors in daily life” (p. 448) and thereby serve as an avoidance mechanism. This view is supported by the present findings and might lead to the assumption that DD such as DID and DDNOS may develop as a result of well-learned and persistent dissociative reaction tendencies. This supposition is consistent with the idea of Nijenhuis and colleagues (2002) who argue that mental avoidance represents avoidance of “emotional personality” (EP) by the “apparently normal personality” (ANP).

Our finding that PTSD patients with DD reported longer and more psychotherapeutic treatments is in line with previous notions that, given the complexity of symptoms, the diagnosis of DID is usually made in the course of longer time periods of therapy (Gast, Rodewald, Kersting, & Emrich, 2001; Gleaves, 1996; Putnam, 2003). This pattern of results can be understood in two ways. On the one hand, our results are consistent with the posttraumatic theory of dissociation (e.g., Gleaves, 1996; Putnam, 2003) and support the idea that a failure to diagnose DD correctly partly accounts for the severe impairment and high use of therapeutic and medical treatments seen in these patient populations. Previous studies have shown that patients with major DD show high use of medical and psychotherapeutic services (Rodewald & Gast, 2005).

Alternatively, the duration and number of therapeutic treatments could be a cause for the development of dissociative symptoms (Piper & Merskey, 2004a, b). With increasing duration of therapy, the evocation of dissociative symptoms in patients who are receptive to suggestion or manipulation by the therapist would be more likely. Even though there is no empirical evidence, yet, that DD patients are more prone to suggestion or manipulation than those without DD (e.g., Dalenberg et al., 2012), one could assume that avoidance tendencies could as well have been suggested in therapy. In fact, the duration and number of therapeutic interventions have been discussed as causes for the development of dissociative symptoms (Piper & Merskey, 2004a, b). With increasing duration of therapy, the evocation of dissociative symptoms in patients who are receptive to suggestion or manipulation by the therapist would be more likely. Similarly, it is not implausible to assume that higher avoidance in patients with co-occurring DD is a side effect of therapeutic interventions.

Even though our findings are encouraging, they should be interpreted carefully as we did not assess quality of early and later parental relations, attachment types and emotional support. These are factors that can buffer or worsen the experience of a potentially traumatizing event. The current study is also limited by a very small sample size; therefore, caution must be applied to the interpretation and generalizability of findings. Thus, our results might be affected by type 2 errors. Even though effect sizes validate the absence of differences between both groups to some extent, the presence of non-differences cannot be verified by means of statistical testing. Moreover, since all participants were inpatients we cannot rule out, that they had a much higher level of psychopathology than those in outpatient treatment, leading to an absence of group differences. However, given that no study has systematically investigated these diagnostic groups so far, the present findings might help to better understand the complex relationship among trauma, PTSD, dissociation, and DD. Future studies are recommended to compare large numbers of patients with PTSD, those with DD, and ideally patients with both PTSD and DD also. Major clinical implications regard possible avoidance tendencies of PTSD patients with co-occurring DD. As discussed above, larger avoidance tendencies may results in longer therapy or longer therapies could be associated with a higher probability to induce avoidance in the patient. As a consequence, interventions should try to address possible avoidance strategies of these patients. Moreover, therapists should be aware of their patient′s disposition to develop and get engaged in avoidance behavior.

In conclusion, bearing in mind the limitations of the present study, our results show that PTSD and co-occurring DD are similar with respect to the overall severity of trauma symptoms as well as comorbid features. In addition, both diagnostic groups were comparable on a variety of characteristics related to trauma history therefore suggesting that they might share a common etiology. The fact that co-occurring dissociation was specifically linked to increased trauma-related avoidance as well as to longer therapeutic treatments might support the idea that DD may reflect a well-learned and automated avoidance mechanism in response to stressful and overwhelming trauma symptoms. This might result in a particularly complex overall presentation of psychopathology with higher levels of impairment that should be addressed by adequate therapeutic approaches taking into account both the similarities but also the differences in treating PTSD only as compared to PTSD and a co-occurring DD.
